# An 8-Fold Parallel Reactor System for Combinatorial Catalysis Research

**DOI:** 10.1155/JAMMC/2006/56329

**Published:** 2006

**Authors:** Norbert Stoll, Arne Allwardt, Uwe Dingerdissen, Kerstin Thurow

**Affiliations:** 1 Institut für Automatisierungstechnik Universität Rostock Richard-Wagner straße 31 Rostock 18119 Germany; 2 Center for Life Science Automation (CELISCA) Friedrich-Barnewitz straße 8 Rostock 18119 Germany; 3 Leibniz-Institut für Katalyse e. V. Universität Rostock} (Auβenstelle Berlin) Richard-Willstätter straße 12 Berlin 12489 Germany

## Abstract

Increasing economic globalization and mounting time and cost pressure on the development of new raw materials for the chemical industry as well as materials and environmental engineering constantly raise the demands on technologies to be used. Parallelization, miniaturization, and automation are the main concepts involved in increasing the rate of chemical and biological experimentation.


Hindawi Publishing Corporation
Journal of Automated Methods and Management in Chemistry
Volume 2006, Article ID 56329, Pages 1–9
DOI 10.1155/JAMMC/2006/56329

### An 8-Fold Parallel Reactor System for Combinatorial Catalysis Research

**Norbert Stoll,[1] Arne Allwardt,[2] Uwe Dingerdissen,[3] and Kerstin Thurow[2]**

[1] *Institut für Automatisierungstechnik, Universität Rostock, Richard-Wagner straße 31, 18119 Rostock, Germany*
[2] *Center for Life Science Automation (CELISCA), Friedrich-Barnewitz straße 8, 18119 Rostock, Germany*
[3] *Leibniz-Institut für Katalyse e. V., Universität Rostock (Außenstelle Berlin), Richard-Willstätter straße 12, 12489 Berlin, Germany*

Received 15 February 2006; Accepted 3 April 2006

Increasing economic globalization and mounting time and cost pressure on the development of new raw materials for the chemical industry as well as materials and environmental engineering constantly raise the demands on technologies to be used. Parallelization, miniaturization, and automation are the main concepts involved in increasing the rate of chemical and biological experimentation.

Copyright © 2006 Norbert Stoll et al. This is an open access article distributed under the Creative Commons Attribution License, which permits unrestricted use, distribution, and reproduction in any medium, provided the original work is properly cited.

### 1. INTRODUCTION

The last few years have seen a dramatic increase in the importance of systems that allow reactions with reactant gases such as hydrogen, oxygen, and carbon monoxide at varying pressure levels. Accordingly, newly developed equipment boast increased reactions per time unit, reduction in reaction volume to be accommodated by ever more compact equipment designs, and increased levels of automation [1, 2].

### 2. CURRENT STATE OF TECHNOLOGY

Currently, there are three conceptually distinct system approaches in reaction technology—single, parallel, and microreaction systems. Another distinguishing feature of each system is its area of application, which is limited to either heterogeneous or homogenous catalysis. Single-reaction systems only have one reaction vessel. Examples are HITEC-Zang LabKit, HEL Auto-Lab, and reactors manufactured by Parr, Premex Reactor, or Büchi. These enable immediate influence on reaction parameters such as pressure, temperature, stirring intensity, and substance concentration. Controlled and directed changes in reaction parameters provide the possibility of reducing the number of experiments, with the elimination of unusable test results cutting research times.

The main advantage of parallel reaction systems is the large number of reaction vessels that allow simultaneous reactions at low pressure (Chemspeed ASW2000, HEL ChemScan lp, Argonaut Advantage Series 3400) or high Amtec SPR16, Argonaut Endeavor System, Symyx PPR, Chemspeed SLT100, HEL Chem-Scan hp). Low-pressure systems usually use a common gas supply, which means that individual pressure adjustment in each reaction is not possible, whereas most reactors that work at pressures above 10 bar are equipped with individual reactor vessel pressure regulators. They not only support the individual parameterization of each reaction vessel, but also provide substance supply under reaction pressure enabling a controlled reaction start. Highly parallelized high-pressure systems such as Symyx HPR, HEL CAT96, celisca HPMR 50–96, Premex Reactor 96x multireactor use a central gas supply, like the low-pressure parallel reactors.

The third group consists of microreaction reactors such as Ehrfeld Mikrotechnik AG, IMM-Mainz GmbH, Syrris Africa. Their main advantage is the large reaction surface that decreases the time taken in the reaction compared to reactors operated in batch mode. Since microreactors use one stirrer, they operate in continuous mode. Their compact size allows for steep pressure and temperature gradients.

The low continuity in reactor volume becomes clear from parallel reaction systems that operate at reaction pressures above 10 bar; the devices on offer mostly have two-figure milliliter volumes, while the 2–3 ml volume range is only seldom covered (Chemspeed SLT 100, HEL CAT96).

### 3. ASSIGNMENT DESCRIPTION

The most important catalytic processes in classical bulk chemistry include high-pressure processes with gases such as hydrogen and carbon monoxide. Up to now, these processes have only been sporadically used in materials development and modification. Performing hydration, carbonylation, and other related reactions under pressure (20–100 bar in all cases) together with the safety measures while providing conditions meeting equipment and safety standards requires the development of suitable reaction systems [3].

Advances in automated reaction technology have shown a marked development in miniaturization in the reaction approach. One factor that has made this development possible is the refinement of analysis conditions. The most important reasons for this development are, firstly, the reduction in substance quantity required and therefore the experimental costs involved, and, secondly, the increase in the number of parallel reactions running relative to the working area available due to miniaturization. This leads to an increased number of reactions in a given time, therefore increasing productivity. However, the more compact reaction systems often need to be adapted to conventional reaction systems with respect to area-to-volume ratios to make future upscaling to larger reaction systems possible at a later stage. This especially applies to catalytically controlled reactions with gases [4].

Often, the most important factor in these heterogeneously or homogeneously catalysed reactions is the exclusion of moisture and/or oxygen. This makes planning for a protection gas system essential when constructing the corresponding equipment. Other parameters that should be covered in a corresponding reactor system are stability under pressures of up to 150 bar and constant operation at temperatures between $-20$ and $+150^\circ$C. Additional requirements are chemical resistance and avoidance of contamination effects [5].

### 4. 8-FOLD PARALLEL REACTOR CONCEPT

The reactor system is based on an arrangement of up to three reactor arrays, each with eight individual autoclaves based on a modular design [6–8]. The concept is aimed towards accommodating individual magnetic stirring systems for each reactor and individual pressure control at pressures up to 150 bar. The system temperature is to be controlled for each array using a circulation thermostat.

The control concept is to consist of a hierarchically arranged distributed system with separated functionality and remote client connectivity. This will be based on a distributed arrangement of microcontroller components connected using a CAN field bus and a higher-level control computer (Figure 1).

### 5. HARDWARE SOLUTION

### 5.1. *General description*

The base modules are made of stainless steel in order to preserve their chemically inert qualities. Apart from the gas supply, they also have a pipette opening to enable the addition of air-sensitive and moisture-sensitive reagents under inert conditions. A third connection serves the reaction vessel also made of stainless steel, where the catalytic processes take place. The rotationally symmetric vessel has an inner volume of 3 ml, emphasizing the main aim of miniaturization in the construction design. The head of the vessel is shaped in such a way as to simplify sample processing by automated analysis.

The reagents are homogenized using magnetic stirrers driven by an integrated mobile stirrer unit installed underneath the reactor array. This is housed in a casing with a design based on a modification of a conventional aluminium heat sink. This ensures that there is sufficient heat dissipation from the coil-based magnetic stirrer drives despite their position near the temperature-controlled reactor.

The temperature is controlled by transporting the heating fluids through drilled openings in the reactor modules that form a closed circuit in combination with their respective end pieces. This ensures a very even temperature distribution. The circulation thermostat combined with DW Therm fluid provides a temperature control range of $-40$ to $200^\circ$C. The heating and cooling rates are $4.5^\circ$C and $2.5^\circ$C per minute, respectively, in temperatures ranging between $25^\circ$C and $100^\circ$C.

Each autoclave is connected to a supply system for inert and process gas, which also purges the system when connected to a vacuum supply.

The respective processes are automated using the integrated measuring and actuation devices. The actuation system is solely based on switch-over valves.

These enable pressure control to an accuracy of under $\pm 0.1$ bar at target pressures of up to 150 bar. The 150 bar pressure limitation is due to the maximum operating pressure of the FAS Bacosol magnetic valves used. Setting the pressure in the eight reactors only takes five minutes. The control principle applied would still allow for the use of other components, thus allowing an extension of the pressure range.

Parker NOVA pneumatic membrane valves are used to seal off the reactor area from the supply system independently of pressure gradient.

The whole system is housed in a compact casing allowing simple integration into standardized laboratory extractor systems.

The results from the catalytic processes are returned to the control system to determine reactor pressure. This involves pressure sensors integrated into the supply system, thus providing continuous information on gas consumption.

### 5.2. *Autoclave array*

The central unit of each array consists of a reactor block consisting of eight reactors made of V4A steel 1.4571 arranged inline in a block. The other parts of each reactor are connected up to the supply system via the gas supply. The interchangeable reaction vessels with a volume of 3 ml are connected into the reactor from underneath and are fixed into the reactor using a vessel nut. These are also made of stainless

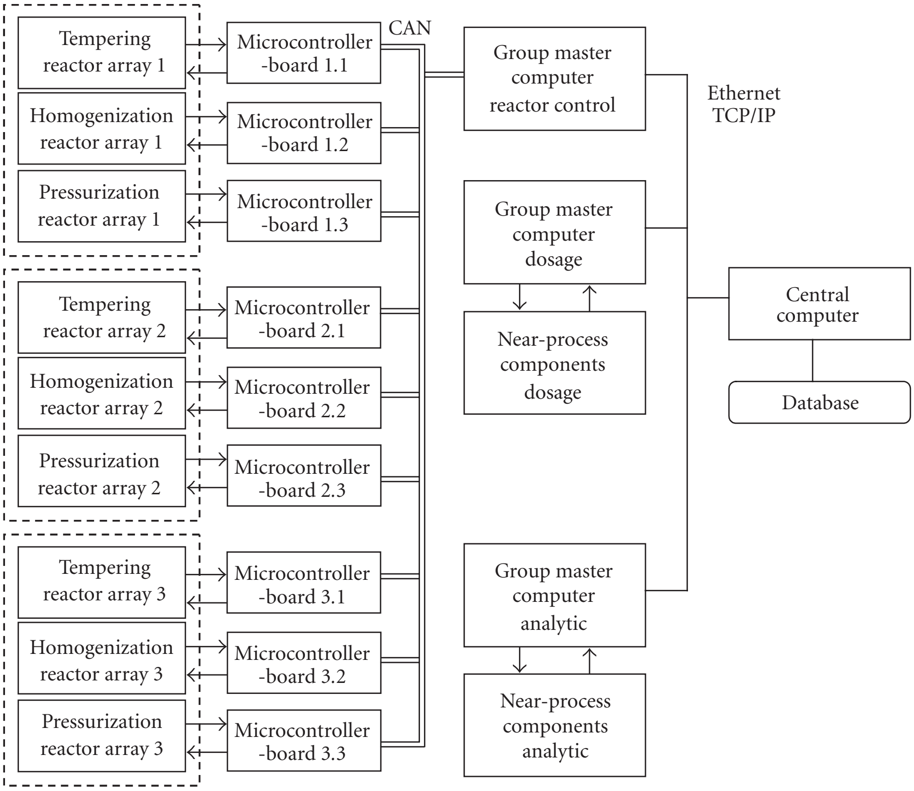

Figure 1: Control concept.

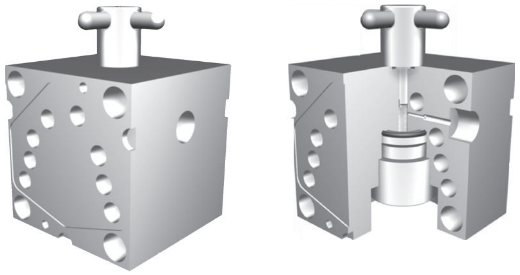

Figure 2: Individual reactor design.

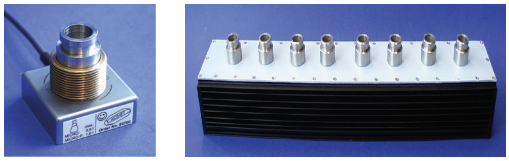

Figure 3: Stirrer unit.

steel 1.4571. A Viton O-ring is used to seal off the reactor and the reactor vessel. This material ensures a broad range of chemical resistance properties. There are drilled openings in the reactor blocks for the silicon oil-based temperature control fluid for the circulation thermostat. The fluid reagents are dosed into the reaction vessels via an opening on the upper side of each individual reactor that can be sealed off using a screwed connection and ring gasket (Figure 2).

### 5.3. Temperature control

The reactor temperature is regulated by an external circulation thermostat that heats or cools the fluid in cavities constructed for the purpose, and pumps the fluid into the reactor block. The circulation thermostat used here is a Huber Kältemaschinen GmbH Unistat Tango device, which is designed as a closed system and ensures a temperature range between −40 and 200°C [9].

The Unistat Tango has an RS232 interface and a controller unit connected to the local control system using a proprietary protocol [10].

### 5.4. Homogenization

Under the reactor a stirrer assembly is arranged in such a way that it can be moved towards the rear, allowing the reaction vessels to be mounted into the reactor block from underneath. The stirrer assembly ensures that the reagents are mixed effectively, which takes place under the control of VARIOMAG MICRO stirrer drives, which drive the magnetic stirrers in the reaction vessels in a rotating field (Figure 3).

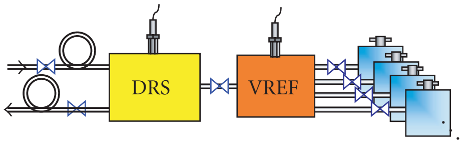

Figure 4: Basic reactor system design.

Each reaction solution can be agitated simultaneously at varying speeds. The stirrers are equipped with a soft-start mechanism in order to optimize start-up. The stirrer unit is dimensioned in such a way that the substances are mixed effectively even under extreme reaction conditions at temperatures of up to 200°C and pressures of up to 200 bar. The stirrer unit is cooled by compressed air to prevent overheating. This assembly is controlled via a CAN interface. Various LEDs show the state of the stirrer unit visually [11].

### 5.5. Gas supply system

The mechanical design of the reactor ensures excellent operating safety at operating pressures above 150 bar. The centralized gas supply system uses conventional fluid automation systems (FAS) two-way valves, and can supply with the reactor with reactive gas, inert gas, or a vacuum for purging (Figure 4).

The individual reactor modules are connected by separately controlled pneumatically actuated NOVA membrane valves with a reference volume. Information can be gained on the inner pressure of the reactor, and therefore the stage of the reaction, from the sequential pressure equalization processes between the valve and each reactor.

The reference volume is connected to a pressure regulation line, which is also used in the gas supply for the system, for which it has inlets for process and inert gases as well as outlets for connection to a vacuum supply and air extraction system.

The detailed reactor structure results from integrating the various actuation and sensor components involved in the basic design (Figure 5).

The individual modules in each reactor block equipped with a temperature sensor (Pt100) are separated from the reference volume by membrane valves, which have an integrated high-accuracy PMP 4070 pressure sensor (0.04% of the end value). This sensor is intended for recording pressure equalization processes. Pneumatic actuators are also used for blocking the reference volume to the pressure regulation line. The target pressures for each individual reactor are set in the pressure regulation line. The corresponding pressure values are measured using a PMP 1400 sensor. The temperature in the pressure regulation line is measured by a Pt100 sensor element.

The connected vacuum system can only be blocked using a conventional FAS Bacosol magnetic valve set in one operating correction due to the constant pressure gradient. A vacuum sensor (Norgren GAH 40 level controller) is used for recognizing potential supply outages in this area.

The pressure is controlled using magnetic valves selected for their dynamic characteristics. The valves are additionally connected using capillary tubing with an inner diameter of 0.5 mm to limit the flow. The other path installed into the outlet branch serves towards slow depressurization of the reaction area and is fitted with a capillary tube with a nominal diameter of 0.25 mm. Another magnetic valve prevents contamination of the pressure regulation line with the two capillary-combined output valves set against their operating direction.

Two more passive elements are integrated into the branches serving the gas supply. These consist of particle filters with a filter range of 5–9 $\mu$ and ball check valves with a trigger pressure of 0.14 bar. The valves are to protect the actuators from excess pressure acting against their operating direction.

Specialized PMP 1400 sensors are integrated into the gas feed of the reactor to ensure real-time process monitoring. The sensors are positioned horizontally over the reference volume. The digital signal conversion electronics is housed in the terminal box already fitted.

The dimensions of the 8-fold parallel reactor are $57 \times 65 \times 30$ cm. The other reactor arrays can be mechanically connected with additional connecting equipment.

### 5.6. Hardware reactor control

The parallelized reaction platform is automated using a distributed control system consisting of intelligent microcontroller units networked using a field bus protocol based on the CAN bus system to a top-level control computer.

Each of the decentralized controller units fulfils a complete control function. The functionality of the DCS is arranged into temperature control, homogenization, and pressure management. In the finished system, two of the microcontroller units were placed in a central electronic equipment rack that also accommodates the power supply units. Figure 6 shows the front and rear of the "19" rack unit. Each unit has at least one front-side status LED for information on operating status. Errors can also be shown visually on the controller units.

Figure 7 shows the structure of the system at process level.

### 5.7. Software reactor control

The system software consists of three separate function modules.

One of these modules is the control software in the electronic units, and governs the microcontrollers that control the hardware to ensure smooth operation in the subprocesses to be carried out. Apart from controlling the actuators, the functions of this software module include reading sensor information and, if necessary, throwing a unit-specific error.

The two other software modules presenting the local control system use the PC as their platform. It is important to distinguish the server used as the system service, which is

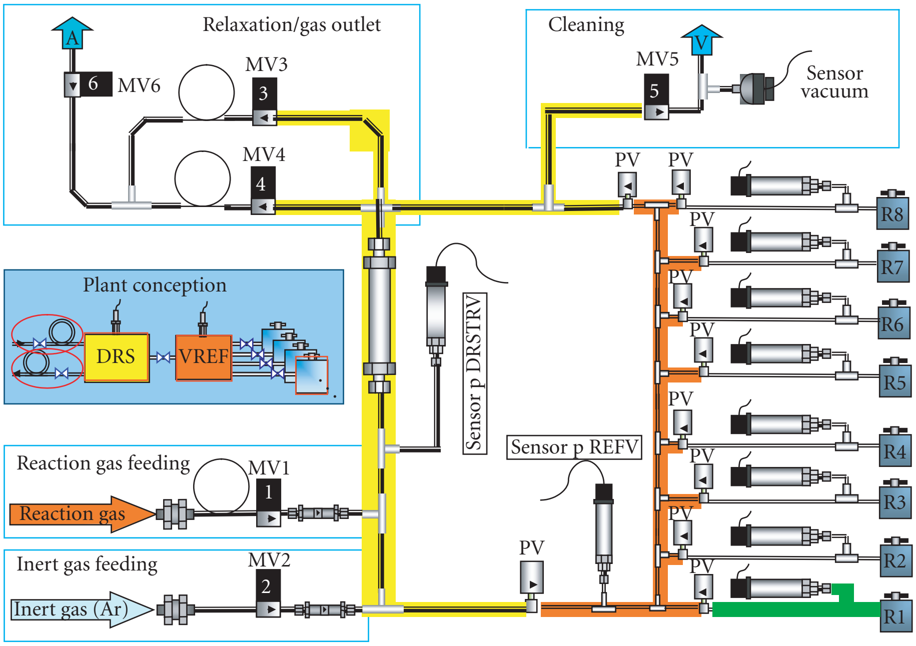

FIGURE 5: Detailed reaction system equipment structure.

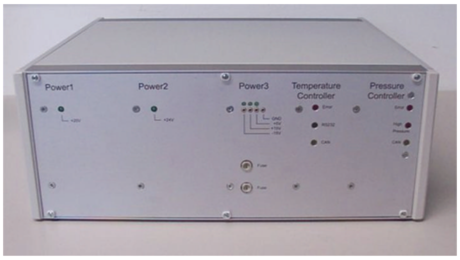

FIGURE 6: Electronic equipment rack.

mainly responsible for data transfer, and the client, which handles visualization, operation, and protocol logging.

The server runs on the computer that is connected to a maximum of three electronic equipment rack units. The three client applications responsible for visualizing the individual 8-fold parallel reactor blocks can be connected to this computer. Data transfer between the reactor control and the local control system uses the CAN field bus. On the other hand, data is transferred between the client and server via the network using an IP protocol.

The actual user interface is provided using complex control software enabling control of the three autoclave arrays—or 24 individual autoclaves.

### 5.8. Local control system

The local control system handles planning, control, and monitoring of automated reaction processes in an 8-fold autoclave array.

The reaction can be halted manually at any time. The control system reacts to any errors that have occurred by halting the reaction.

The field bus is used for communication with the autoclave array using a proprietary protocol.

An LIM system can be used for planning and starting the reaction.

The user interface ensures user-friendly chemical reaction operation. This requires data transfer with the server using a TCP/IP-based interprocess communication protocol. The reactor data are logged to a protocol file for later review. An export function enables subsequent processing and analysis of the log data using, for example, MS Excel.

The functional emphasis of the system mainly lies in the following areas:

(i) visualization and operation;
(ii) process management and control;
(iii) archiving and protocol logging;
(iv) process communication.

The control software is Windows-based and has a two-part design corresponding to the client-server model. The clients are responsible for the visualization, process management, and logging function while the server manages time-critical communication with the distributed units at process level. In order to ensure at least close to real-time conditions, this part of the software runs as a high-priority system service [12, 13].

Communication between the two software parts is based on a TCP/IP protocol to ensure remote communication between the server and client. The client is responsible for visualization and runs a typical Win32 MDI (multiple-document interface) application that can handle a number of documents at the same time.

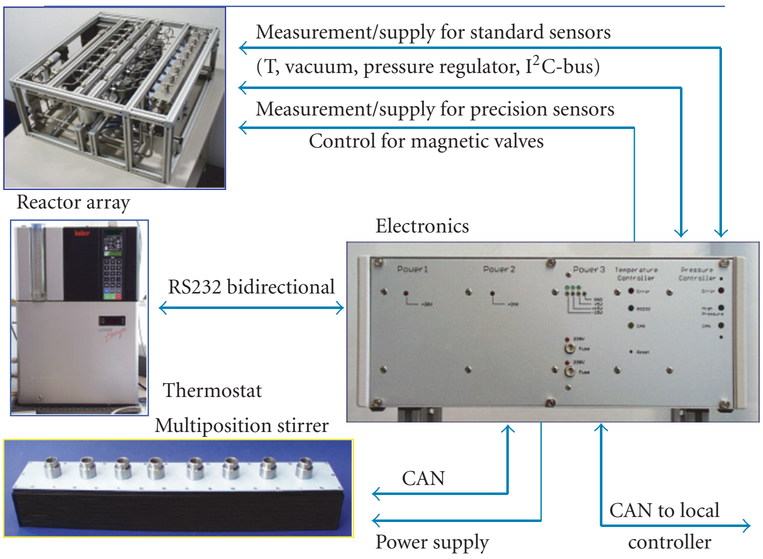

Figure 7: Local control system.

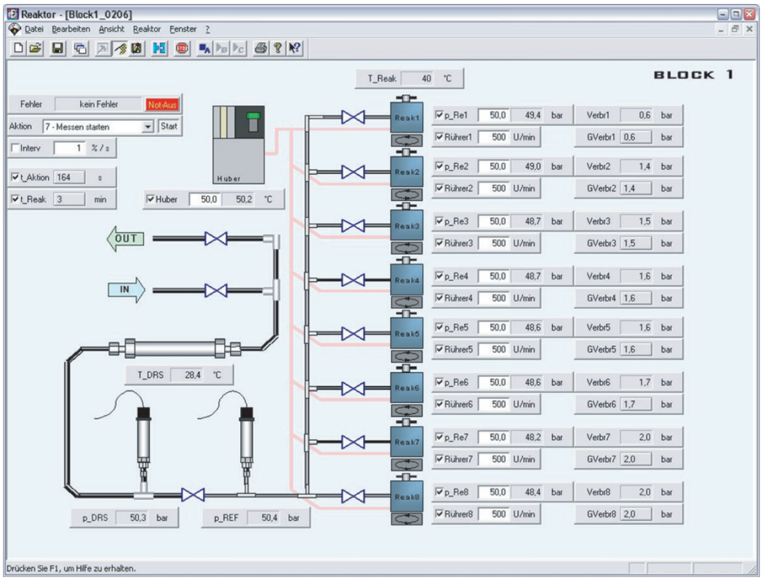

Figure 8: Control software and reactor control document layout.

### 5.9. *Visualization and operation function*

There are two document types. One of their functions is to visualize the state of the reactor system and its operation, and the other is to edit the command lists for automatic experiment control.

The document for operating the reactor system is run as an OLE container, and its appearance can be altered according to the preferences of the end user. The layout consists of a background (an image or graphic) and a number of visualization and data-entry elements that can be placed as required. Figure 8 shows the default configuration for operating a reactor array, which is modeled on the systematic structure of the actual system.

Global operation takes place using the controls at the top left-hand part. Control commands can be sent to the pressure management unit using the drop-down menu (“Aktion” control). The system status visualization is also shown here.

The target values can be entered into the corresponding entry fields. The respective actual values are shown as numbers. There is also the possibility of displaying the progress of each value of any control element in a diagram, allowing the

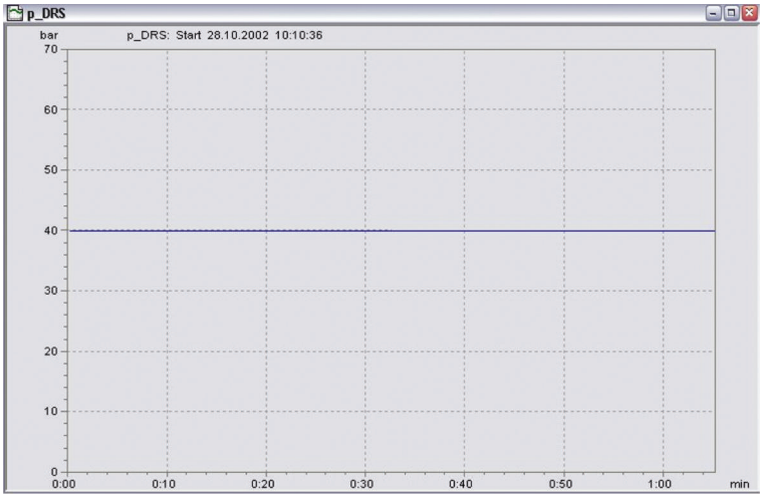

Figure 9: Diagram representation.

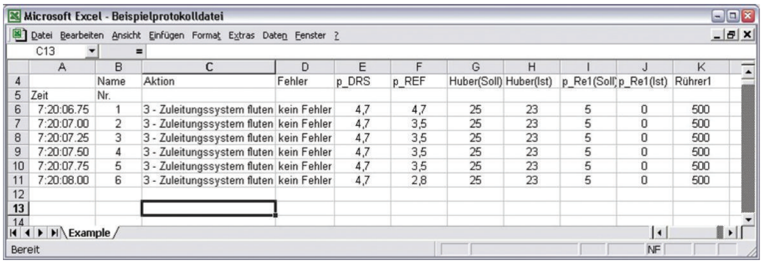

| | A | B | C | D | E | F | G | H | I | J | K |
|---|---|---|---|---|---|---|---|---|---|---|---|
| 4 | | Name | Aktion | Fehler | p_DRS | p_REF | Huber(Soll) | Huber(Ist) | p_Re1(Soll | p_Re1(Ist) | Rührer1 |
| 5 | Zeit | Nr. | | | | | | | | | |
| 6 | 7:20:06.75 | 1 | 3 - Zuleitungssystem fluten | kein Fehler | 4,7 | 4,7 | 25 | 23 | 5 | 0 | 500 |
| 7 | 7:20:07.00 | 2 | 3 - Zuleitungssystem fluten | kein Fehler | 4,7 | 3,5 | 25 | 23 | 5 | 0 | 500 |
| 8 | 7:20:07.25 | 3 | 3 - Zuleitungssystem fluten | kein Fehler | 4,7 | 3,5 | 25 | 23 | 5 | 0 | 500 |
| 9 | 7:20:07.50 | 4 | 3 - Zuleitungssystem fluten | kein Fehler | 4,7 | 3,5 | 25 | 23 | 5 | 0 | 500 |
| 10 | 7:20:07.75 | 5 | 3 - Zuleitungssystem fluten | kein Fehler | 4,7 | 3,5 | 25 | 23 | 5 | 0 | 500 |
| 11 | 7:20:08.00 | 6 | 3 - Zuleitungssystem fluten | kein Fehler | 4,7 | 2,8 | 25 | 23 | 5 | 0 | 500 |
| 12 | | | | | | | | | | | |
| 13 | | | | | | | | | | | |
| 14 | | | | | | | | | | | |

Figure 10: Example of an exported file.

user to follow the reaction trends in real time. Figure 9 shows an example of this diagram in which both the time and value axes are scalable.

### 5.10. Process management and control

Process management and control can take place on two levels. In the first, the user can influence the reaction manually using a mouse and keyboard on the layout interface described above. In the second level, the system allows automated reaction management using an editable command list, which can implement processes based on time or event. This sets the basis for carrying out reproducible experiments.

The list can be shown and edited using the second document type that the control software supports. The list can be edited using logically linked drop-down menus.

Command list creation is simplified by an additional macrorecording function. This allows the user to record the commands carried out on the software control layout, which are then stored as a time-based command list. Note that only time-based processes can be programmed using this functionality. The user will have to use the drop-down menus to enter limit value and conditional statements as well as branching instructions.

### 5.11. Archiving and protocol logging

The values and events displayed on the interface are logged by the local control system. This functionality is specifically provided by the client software. All data created is recorded every 250 ms into a protocol log file with the rpf extension. A new file is created every day and carries the current date and the computer name of the server used in its filename.

The client has an export function that can export the rpf file into a format that can be read by spreadsheet software. The export process is controlled using menus on the client, and allows the user to select certain data, time intervals, and periods (as multiples of the basic interval). The result is a tab-separated text file in ASCII format that can be read by any DOS-based system, and can be opened and processed by standard data processing packages such as MS Excel or MATLAB (Figure 10). Therefore, the process data logged can also be used after the experiment to analyse the chemical reaction course.

### 5.12. Process communication

The distributed microcontroller units and the higher-level control system structures have to be able to communicate.

The control system is connected to the process level units using the server application and a CAN interface. The latter is managed using a software library supplied by the manufacturer that gives the system the corresponding driver function [12]. Status information on each process unit is read at 250 ms intervals.

The higher-level control level is connected using the client software and a TCP/IP protocol. A script interface allows the user to enter target values, read actual values, and execute command lists. This enables the integration of the reaction equipment into the whole automation system with other dosing and analysis function units.

### 6. OPERATING MODES

The reactor can be operated in two operating modes—isochoric and isobar modes.

### 6.1. Isochoric mode

The sensors that directly measure the inner pressure are read at set intervals, thus delivering current information on the actual reaction process. Due to the characteristics of the multichannel analog-digital converter and the input circuit, the operator has to wait for a constant value reading after switching channels. A measuring interval of 200 ms has been selected that leads to a total running time of 1.6 s taking array dimension into account.

The measuring process takes place throughout the entire reaction period and is directly connected to the filling system, which is made possible by the integration of high-pressure sensors in the compressed gas system for each individual autoclave.

The decreasing pressure during the reaction gives information on the gas consumption in the reaction.

### 6.2. Isobar mode

The advantages of isobar mode operation are the constant pressure conditions during the reaction. The user has to define a maximum permissible pressure decrease in the reactors. If the pressure falls below a certain threshold value, a sequence is triggered that adjusts the pressure back to the target pressure via the supply system.

### 7. CHEMICAL APPLICATION SYSTEM TEST: CARBONYLATION

### 7.1. Introduction to the reaction

The system was tested with catalytic carbonylation reactions using a carbon monoxide supply. The reaction selected for determining precision and screening reaction parameters was carbonylation of iodobenzene to methylbenzoate using a palladium catalyst (see Figure 11) as described in the literature [14].

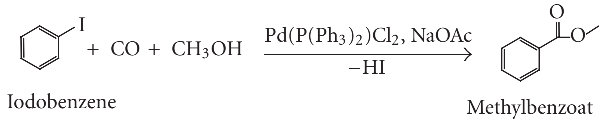

Figure 11: Reaction diagram for carbonylation of iodobenzene to methylbenzoate.

Table 1: Example of an exported file.

| Reactor | 6 h at 60°C | 1 h at 80°C |
|---|---|---|
| | Rate in % | Rate in % |
| 1 | 79.40 | 75.77 |
| 2 | 78.14 | 74.05 |
| 3 | 74.87 | 79.45 |
| 4 | 80.22 | 76.78 |
| 5 | 79.59 | 77.49 |
| 6 | 81.03 | 75.35 |
| 7 | 80.37 | 77.49 |
| 8 | 80.10 | 83.74 |
| Average | 79.216 | 77.516 |
| Standard deviation, abs. | 1.951 | 2.993 |
| Standard deviation, rel. | 2.463 | 3.862 |

### 7.2. Examination of precision under comparison conditions

To examine the precision under comparison conditions, all eight reactors were filled with identical reaction solutions, and the contents of each reactor were exposed to the same reactor conditions (60°C temperature and 6 h reaction time, or 80°C temperature, 1 h reaction time, H2 starting pressure: 50 bar, stirrer speed: 300 rpm). The results are summarized in Table 1.

In contrast to the reports from the literature stating that a reaction temperature of 60°C at a pressure of 200 psi (14 bar) and a reaction time of 48 h should yield a rate of 80%, this rate was already reached after 6 h at a reagent gas pressure of 50 bar, and 60 min after increasing the temperature to 60 min. At relative average standard deviations of 2.46% and 3.86%, the values were reached under comparison conditions were present.

### 7.3. Screening examinations

Screening experiments with variations in the following reaction parameters were carried out to demonstrate the power of the octuple parallel reactor system:

(i) temperature: 60–90°C;
(ii) CO starting pressure: 10–40 bar;
(iii) stirrer speed: 100–900 rpm;
(iv) reaction time: 1–3 h.

Figure 12 shows a graphical representation of values reading using double determination by varying reagent gas pressure

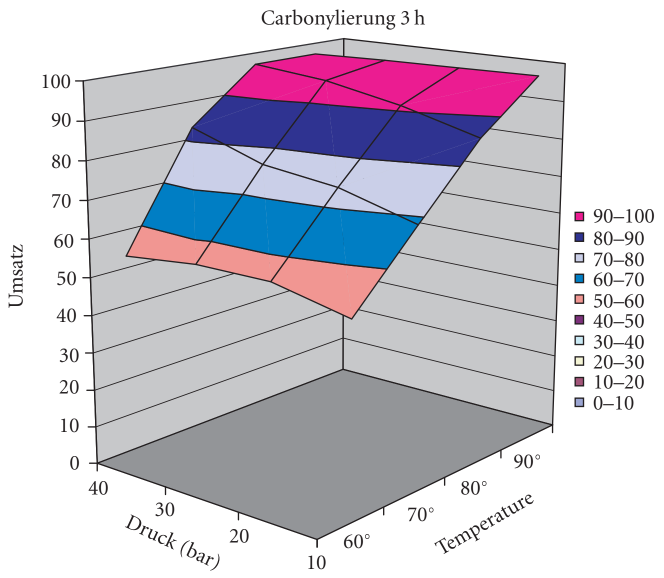

Figure 12: Summary of results for a 3 h reaction period, 300 rpm stirrer speed.

and reaction temperature at a reaction period of 3 h and a stirrer speed of 300 rpm.

The graphic shows that the maximum of the range examined is already reached at a temperature of 80°C and reagent pressures between 30 and 40 bar.

### 8. SUMMARY

As the results show, the octuple reactor is a parallelized system that can be used for various reactions with gases under high pressure at volumes ranging from 1.5 to 2 ml. With inert gas conditions ensured, air-sensitive substances can also be used and several reaction parameters can be optimized at the same time.

Development of various septum adapters will allow substance injection at higher pressures into the reactor.

### REFERENCES

[1] B. T. Haag, "Automated and unattended parallel synthesis integrating work-up and analysis," *Chimia*, vol. 54, no. 4, pp. 163–164, 2000.

[2] Frost & Sullivan (Hrs.), *Combinatorial Chemistry: Powerful New Tool for Discovery and Product Development*, Marktreport, New York, NY, USA, 2nd edition, 2002.

[3] Y. K. Yun and J. Labadie, "Parallele katalytische Hydrierung fokussierter Verbindungen," *GIT Laborzeitschrift*, vol. 44, no. 12, pp. 1456–1458, 2000.

[4] G. Jung, *Combinatorial Chemistry*, VCH Verlagsgesellschaft mbH, Weinheim, Germany, 1999.

[5] F. A. Rampf and W. A. Herrmann, "High-Throughput-Katalysatorscreening unter Hochdruckbedingungen," *Chemie Ingenieur Technik*, vol. 73, no. 1-2, pp. 97–99, 2001.

[6] K. Thurow, A. Allwardt, C. Wendler, and N. Stoll, Kombinatorische Methoden zur effizienteren Entwicklung von Katalysatoren - Teilprojekt Automation, Schlussbericht zum Projekt 03C0304B an das BMBF, Universität Rostock 2003.

[7] R. Lemke, "Automation eines miniaturisierten Hochdruckreaktionssystems," Dissertation, Universität Rostock, Rostock, Germany, 2003.

[8] A. G. Degussa, B. E. Bosch, T. Riermeier, et al., Array of Autoclaves, DE 100 49 078 A1 und WO 2002030557 A2, 2002.

[9] Firmenveröffentlichung, Betriebsanleitung Umwälzthermostat Unistat, Huber Költemaschinenbau GmbH, Betriebsanleitung, Offenburg, Germany, 2000.

[10] J. Kozik, Anbindung eines Umlaufthermostaten an das Controller-Area-Network, Strudienarbeit, Universität Rostock, 1999.

[11] A. Meinel, "Entwicklung einer verschleißfreie Mehrpositions-Rühreinrichtung für chemische Reaktoren auf Basis einer Mikrocontrollersteuerung mit CAN-Anbindung," Diplomarbeit, Universität Rostock, Rostock, Germany, 1999.

[12] H. Dahl, "Entwicklung einer programmierbaren Softwareoberfläche für Windows NT 4.0 zur zeit- und ereignisbasierten Ablaufsteuerung eines chemischen Reaktors," Diplomarbeit, Universität Rostock, Rostock, Germany, 1997.

[13] H. Dahl, M. Krohn, S. Junginger, T. Roddelkopf, and N. Stoll, Prozessleitsystem unter Windows NT 4.0 für vernetzte intelligente Komponenten eines dezentralen Mess- und Steuerungssystems, 2. Wismarer Automatisierungssymposium, Wismar, Germany, 1999.

[14] J. K. Stille and P. K. Wong, "Carboalkoxylation of aryl and benzyl halides catalyzed by dichlorobis(triphenylphosphine)palladium(II)," *Journal of Organic Chemistry*, vol. 40, no. 4, pp. 532–534, 1975.

